# Quantitative Analysis of Diffusion Weighted MR Images of Brain Tumor Using Signal Intensity Gradient Technique

**DOI:** 10.1155/2014/619081

**Published:** 2014-05-28

**Authors:** S. S. Shanbhag, G. R. Udupi, K. M. Patil, K. Ranganath

**Affiliations:** ^1^Electronics and Communication Engineering, Gogte Institute of Technology, Belgaum 590008, India; ^2^Indian Institute of Technology Madras, Belgaum 590009, India; ^3^RAGAVS, Diagnostics and Research Center Pvt. Ltd., Bangalore 560011, India

## Abstract

The purpose of this study was to evaluate the role of diffusion weighted-magnetic resonance imaging (DW-MRI) in the examination and classification of brain tumors, namely, glioma and meningioma. Our hypothesis was that as signal intensity variations on diffusion weighted (DW) images depend on histology and cellularity of the tumor, analysing the signal intensity characteristics on DW images may allow differentiating between the tumor types. Towards this end the signal intensity variations on DW images of the entire tumor volume data of 20 subjects with glioma and 12 subjects with meningioma were investigated and quantified using signal intensity gradient (SIG) parameter. The relative increase in the SIG values (RSIG) for the subjects with glioma and meningioma was in the range of 10.08–28.36 times and 5.60–9.86 times, respectively, compared to their corresponding SIG values on the contralateral hemisphere. The RSIG values were significantly different between the subjects with glioma and meningioma (*P* < 0.01), with no overlap between RSIG values across the two tumors. The results indicate that the quantitative changes in the RSIG values could be applied in the differential diagnosis of glioma and meningioma, and their adoption in clinical diagnosis and treatment could be helpful and informative.

## 1. Introduction

Neurological disorders pose a great challenge to healthcare in developing countries, as limited resources and manpower are not enough to tackle the increasing burden [1]. Although brain tumor is not a frequent neurological disorder, still it contributes significantly to morbidity and is no longer rare in clinical practice [2, 3]. Brain tumors can present challenging medical problems, and effective medical management would result in decreased morbidity and mortality and improved quality of life. Brain tumors are generally classified as either primary (originating from within the brain cavity) or secondary (originating elsewhere in the body, and then spread to the brain). The most common types of primary brain tumors are gliomas and meningiomas, constituting, respectively, 60% and 20% of all intracranial tumors (tumors within the brain) in adults [4]. Treatment varies from one tumor type to the other and often involves a combination of surgery, radiotherapy, and chemotherapy. Meningiomas are almost always benign tumors and have good prognosis after surgery, whereas gliomas being malignant tumors comprise multidisciplinary approach and have relatively poorer prognosis. Hence it is very essential to differentiate between the two tumor types especially when gliomas are peripherally placed and imitate a meningioma [4, 5].

The introduction of imaging techniques into clinical practice has been among the most important of all advances in the care of patients with brain tumors. The potential advantage of advanced magnetic resonance imaging (MRI) methods is that they permit more uniform sampling than heterogeneous biopsies, and that, they can be applied repeatedly to monitor therapy success [6]. Diffusion weighted-magnetic resonance imaging (DW-MRI) is an imaging technique based on the sensitivity of magnetic resonance (MR) to microscopic mobility of water molecules within tissues, and provides image contrast that is dependent on the molecular motion of water, which may be substantially altered by disease [7]. DW-MRI probes water molecular diffusion over distances that correspond to typical cell sizes, and this diffusion is impeded by membranes (structures that are an integral part of the cell-architecture) [6]. Of the histologic features for tumor analysis, cellularity has been the target of quantitative assessment with DW-MRI. The rationale of using diffusion imaging to quantify cellularity is based on the principle that water diffusivity within the extracellular compartment is inversely related to the content and attenuation of the constituents of the intracellular space. With increasing cell density, the impeding effect of the membranes is expected to increase. Therefore, tumors with higher cellularity show increased signal intensity on diffusion weighted (DW) images, in contrast to the tumors with low cellularity that presents greater signal intensity loss [8]. Quantification of the amount of diffusion of water molecules in terms of signal intensity variations found within the brain tumors can thereby provide information about the histologic composition and biologic aggressiveness of the tumor [9]. As a result, the variation in the signal intensity observed on DW images for the different types of brain tumors makes the technique helpful in the differential diagnosis of these tumors [10].

Quantification of the amount of diffusion of water molecules found within and adjacent to brain tumors in the biological tissues has been carried out in the past by means of an apparent diffusion coefficient (ADC) that provides information about the tumor cellularity. The higher the tumor cellularity, the lower the ADC because of decreased water diffusivity caused by a relative reduction in extracellular space for the proton to move about [11]. Although the ADC is thought to be inversely correlated with tumor cellularity, and hence tumor grade, its clinical effect remains limited as studies indicate a substantial overlap in the regional ADCs between gliomas of differing grades [11, 12]. Investigators have correlated the ADC values with the cellularity of glial [12] and nonglial brain tumors, such as meningioma [13]. Others have found that in the case of intracranial tumors, high signal intensity on DW-MRI had low ADC values, and further, signal intensities and ADC values appeared significantly different among the tumor types [14]. Additionally, studies have found that ADC values are useful for the differentiation of some human brain tumors [15], and that water diffusivity and the ADC values could be reliably used for tumor differentiation [16]. Although there are several reports in the past that discuss the application of ADC values in tumor analysis [17–23], there are studies that report contradictory findings [24–27]. Most of the previous studies [18, 28–30] are based on selected region of interests (ROIs) placed on a representative section of the tumor for analysis. It is widely known that a single glioma, especially if high grade, may demonstrate a wide spectrum of histologic features; in turn, the ADCs within a given glioma can vary widely between different regions of that tumor. Therefore, an ADC derived from regional ROIs will likely underestimate the heterogeneity of the tumor cellularity [11]. Moreover, the selection of a localized area within a tumor can be subjective and prone to sampling bias [31]. This sampling bias may explain the discrepant results among previous articles [30, 32, 33] for the ability of DW-MRI to distinguish high- from low-grade gliomas. Moreover, these methods depend on correct identification of the measured structure by the operator, and the choice of location, number, and size of the ROI areas is operator dependent. This can lead to site-selection bias, which limits the reproducibility of results [34, 35].

A more objective approach than placement of regional ROIs would be to analyze the entire volume of the tumor to provide quantitative information about the tissue characteristics and heterogeneity of the whole tumor [34]. By encompassing the whole tumor, potential sampling bias would be largely eliminated, and the tumor areas with different diffusion characteristics would be reflected, meaning that all elements of the tumor that contribute to group differences would be analyzed [31]. The ADCs of glioma and meningioma are related to tumor cellularity and thereby DW-MRI and ADCs can provide information useful to diagnose these brain tumors that cannot be obtained with conventional MRI [12]. Although there are reports that qualitatively describe the signal intensity characteristics of glioma and meningioma on DW images [12, 13, 36], quantifying the signal intensity on DW images with whole tumor volume for these tumors is not much reported in literature. However, visual assessment of the relative tissue signal attenuation on DW images is being applied in the clinical practice for tumor detection, tumor characterization, and the evaluation of treatment response in subjects [9]. The purpose of the present work was to evaluate the role of DW-MRI in the examination and classification of brain tumors, namely, glioma and meningioma. An attempt is made to explore the usefulness of the signal intensity characteristics on DW images to differentiate between subjects with gliomas and subjects with meningiomas, based on the entire tumor volume data. The signal intensity variations on DW images are quantified to identify the different levels of changes taking place in the subjects with tumor and further applied in the differential diagnosis of the tumors.

## 2. Materials and Methods

Brain tumors are a heterogeneous group of neoplasms, each with its own biology, treatment, and prognosis. DW-MRI allows noninvasive examination of tumor cellularity since cells constitute barriers that restrict microscopically the motion of the water molecules within the tissue. Increased cellularity in the tumor tissues restricts the regular water movement compared to normal brain tissues and vice versa. In this sense, the diffusion of water molecules across the tumor as compared to the diffusion in normal brain is expected be different, depicting different diffusivity, depending on the tumor cellularity. Thereby, a homogeneous signal intensity variation is observed in the normal region of the brain as compared to the region of tumor, wherein there is a heterogeneous signal intensity variation. Understanding the heterogeneity of individual tumors may prove to be useful in the diagnosis and classification of tumors.

The variation in the signal intensity of different types of brain tumors on DW images yields qualitative and quantitative information that provides unique insight into tumor characteristics. It is observed that the signal intensity of gliomas on DW images is variable (hyper-, iso-, or hypointense). The higher grade gliomas contain greater cellularity and fewer extracellular spaces. In the tissue microenvironment, water diffusion is more constrained, and tissues consequently display greater signal intensity on DW images and low signal intensity on ADC maps [18, 20]. Most benign meningiomas are isointense on DW images and ADC maps. High signal intensity on DW images and reduced ADC values suggest malignant meningiomas [13]. Diffusion restriction in malignant meningiomas is probably due to high tumor cellularity [13]. Thereby by carrying out DW-MRI studies and determining signal intensity variations across the brain tumor area it is possible to distinguish different regions within the tumor and also differentiate between the tumor types. Further, differentiation between these tumor types can play a significant role in planning of therapy and also evaluating response to therapy.

### 2.1. Clinical Data and Diffusion Imaging Protocol

The clinical data is obtained from RAGAVS Diagnostic and Research Center, Bangalore, India, and Vikram Hospital, Bangalore, India. The ethics approval is obtained from the committee of clinical research at the RAGAVS Diagnostic and Research Center and Vikram Hospital to carry out the investigations on the clinical data provided. The study group includes 20 cases (14 males, 06 females; mean age, 55 years; age range, 36–86 years) with clinically proven glioma and 12 cases (04 males, 08 females; mean age, 59 years; age range, 50–78 years) with clinically proven meningioma.

All the subjects underwent clinical MR imaging with 1.5 T Symphony Maestro Class MR scanning system from Siemens. DW-MRI was performed by using a multisection, single-shot, spin-echo, echo-planar pulse sequence with the following parameters: repetition time [TR] = 3200 ms, echo time [TE] = 94 ms, acquisition matrix = 128 × 128, field of view [FOV] = 230 mm × 230 mm, and diffusion gradient value of *b* = 1000 s/m^2^ along 19 axial slices, 5 mm thick slice, and intersection gap of 1.5 mm. For all the subjects with tumor considered in the study, we confirmed the appearance of tumor by ruling out the possibility of bright intensity due to T2 shine through effects. This was done by verifying the appearance of hyperintensities on DW images and concomitant-reduced ADCs relative to the contralateral normal brain in their initial MRI studies.

### 2.2. Signal Intensity Gradient (SIG)

DW-MRI technique makes it to visualize the altered rates of water diffusion, by producing a high signal intensity appearance in the area of tumor, compared to the normal brain tissues. Consequently, on examining the spatial intensity variation distribution on DW images for the subjects with tumors, it is observed that the signal intensity is not uniformly distributed over the entire DW image. There are abrupt jumps in signal intensity in the area of tumor in contrast to the other healthy areas of the brain, where the signal intensity distribution is almost uniform. The gradient of an image measures the rate of intensity changes taking place within the image. The magnitude of the gradient at a particular point within the image provides information on how the image intensity is changing at that point, leading to a higher value for the higher rate of intensity changes and a smaller value for uniform intensity changes. DW images of the subjects with brain tumors, showing bright imaging appearance in the areas of tumor, would thereby lead to higher values for intensity gradients in contrast to the other healthy areas of the brain. In the present work, we have made an attempt to investigate the water molecule diffusion patterns producing signal intensity variations on DW images in the area of tumor and, further, quantify the amount of signal intensity variations using SIG parameter.

### 2.3. Image Analysis

**Figure 1:**
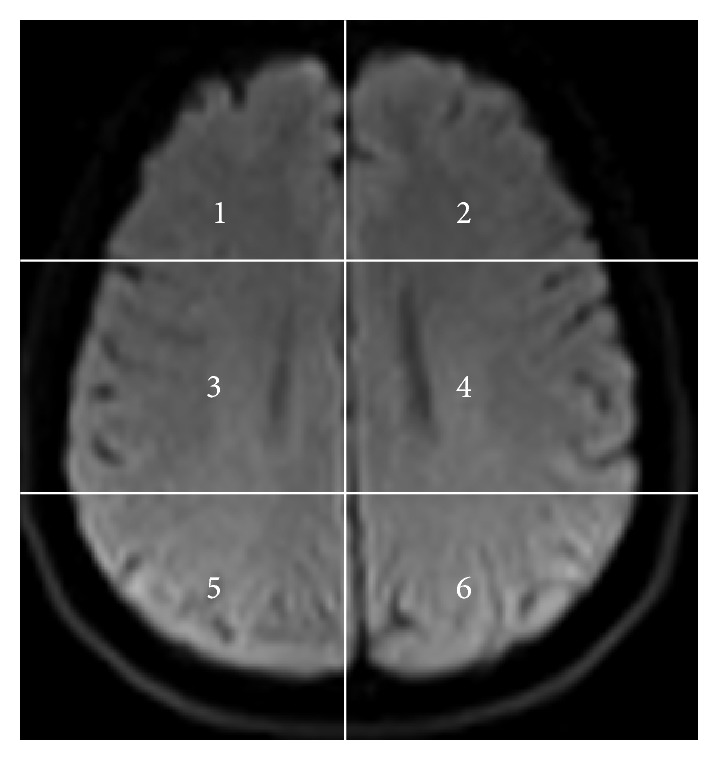
Axial DW image showing areas.

The axial DW images of the subjects with brain tumor, obtained in DICOM format, are converted to bmp format with intensities scaled to fit the conventional range of 0–255. This is done to reduce the complexity in the image manipulation algorithms and to achieve speed up in processing of the images. The axial DW image from each tumor subject is divided into six areas for the convenience of analysis as shown in Figure 1.

**Figure 2:**
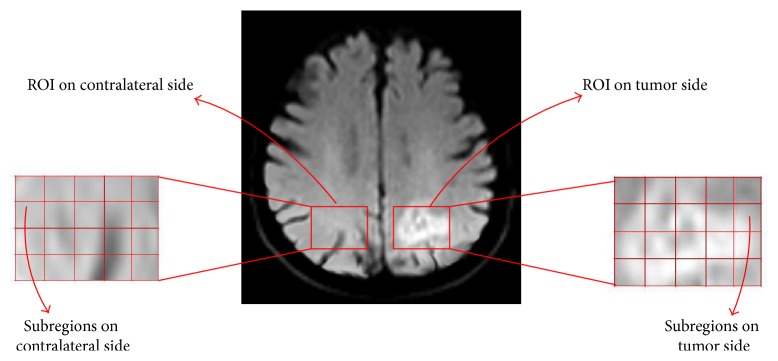
Axial DW image showing ROI (*I*(*x*, *y*)) and subregions (*f*(*x*, *y*)) on tumor side (area 6, axial slice 13) and contralateral normal side.

Each subject with brain tumor is represented by a set of DW images in the axial plane taken at different axial levels (from 1 to 19). The subject with brain tumor presents abrupt changes in the signal intensities in one (or more) of the six areas on the affected axial slices. The axial slices that indicate such abrupt changes in the signal intensities are selected for image analysis. For each axial slice selected from the tumor subject, the following procedure is employed to obtain a decisive SIG value for that subject. To carry out image analysis an ROI, *I*(*x*, *y*), is manually drawn to cover the bright region in the particular brain area, representing the region of tumor. As a control for signal intensity, another ROI of the same size is placed in the same (mirror image) location in the contralateral hemisphere. The ROIs on each side are further divided automatically (using Adobe Photo-shop CS3) into smaller subregions, *f*(*x*, *y*), corresponding to an image size represented by (*M* × *N*) pixels. The typical values for *M* and *N* are chosen in the range between 13 and 18 pixels. Additionally, extra care is taken while choosing the subregions (especially on the tumor side) to avoid any errors that may be caused due to edge effects (change in intensity along the periphery of tumor). The ROI and the corresponding subregions on the tumor side and on the contralateral normal side for a subject with glioma (area 6, axial slice 13) are shown in Figure 2.

**Figure 3:**
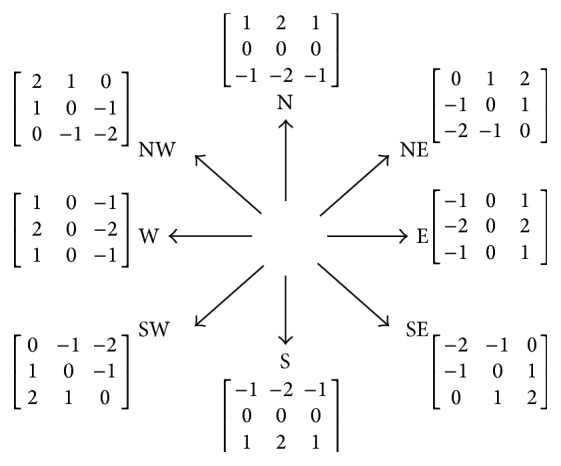
Robinson kernels and their orientation.

Each and every subregion of the ROI on the tumor side is analysed for the variation in signal intensities observed in that subregion. The nature of signal intensity changes in the subregion provides information about the water diffusion characteristics and thereby provides information about cellular density and tumor behavior. Given that water diffusion has directionality, in order to take care of the orientation dependent contrast (diffusion anisotropy), the SIG value for each subregion, *f*(*x*, *y*), is evaluated using the Robinson compass operator, comprising eight kernels, as shown in Figure 3. The set of 8 kernels are convolved with the subregion, *f*(*x*, *y*), to obtain the SIGs along the corresponding directions. G_S_, G_SE_, G_E_, G_NE_, G_N_, G_NW_, G_W_, and G_SW_, represent gradients along South, South-East, East, North-East, North, North-West, West, and South-West directions, respectively. The magnitude of the maximum change in signal intensities can be evaluated as the maximum value gained for the SIG parameter, subsequent to applying all the eight kernels to the pixel neighborhood [37, 38]. The maximum response from all the 8 kernels (SIG_max⁡_) is given by

(1) $$\begin{array}{c} {\text{SIG}}_{\max}=\max\left\{{\text{G}}_{\text{S}},{\text{G}}_{\text{SE}},{\text{G}}_{\text{E}},{\text{G}}_{\text{NE}},{\text{G}}_{\text{N}},{\text{G}}_{\text{NW}},{\text{G}}_{\text{W}},{\text{G}}_{\text{SW}}\right\}. \end{array}$$

The above procedure is repeated for all the subregions of the ROI on the tumor side. The subregion of the ROI that results in the highest SIG_max⁡_ value is the subregion that exhibits highest cellular density and is chosen as the resultant SIG value for that axial DW slice. The resultant SIG value so obtained for the axial DW slice indicates whether there is any sudden change in the signal intensity level in any of the subregions of the ROI for that axial slice.

Subsequently, the subregions of the (mirror) ROI on the contralateral hemisphere are considered for image analysis. The set of 8 kernels are convolved with each subregion to obtain the SIGs along the corresponding directions. However in case of contralateral normal hemisphere, by taking the maximum response from the 8 kernels (as on tumor side), we observed that the SIG values were irregular over the normal hemisphere. They were considerably high especially at the regions of sulci (depression or fissure in the surface of the brain [39]) and gyri (elevated convolutions on the surfaces of the cerebral hemispheres [39]). As an alternative, the minimum response from all the 8 kernels (SIG_min⁡_) is taken as the resulting SIG value for the subregion on the contralateral hemisphere, as it best represents the normal side of the brain (region with uniform signal intensity variation). Eventually, SIG_min⁡_ values are obtained for all the subregions of the ROI on the contralateral hemisphere, and the lowest of all the SIG_min⁡_ values is chosen as the resultant SIG value for the axial DW slice.

The above procedure is repeated for all the subsets of axial DW slices selected for image analysis from the tumor subject. To take into consideration the effect of the presence of tumor on multiple axial DW slices of the subject, the average of the resultant SIG values evaluated across all the axial DW slices is taken as the decisive SIG value for the tumor subject. Similarly, the average of the resultant SIG values from the contralateral hemisphere is evaluated. The decisive SIG value on the tumor side is compared with the corresponding decisive SIG value on the contralateral hemisphere, of the same subject, for quantification of the results using relative SIG (RSIG) value. The RSIG value for each tumor subject is evaluated using

(2) RSIG=(SIG on⁡ contralateral hemisphereSIG on⁡ tumor side hhh.−SIG on⁡ contralateral hemisphere) ×(SIG on⁡ contralateral hemisphere)−1.

Statistical analysis is carried out using excel program and SPSS 17.0 software package. The results are tested for statistical significant difference between the SIG values obtained from the subjects with tumor and their contralateral counterpart and also for the RSIG values across the two tumor types. The analysis is carried out at 99% confidence level (*P* < 0.01) using the significance of difference between the means using Student's *t*-distribution [40].

## 3. Results and Discussions

**Figure 4:**
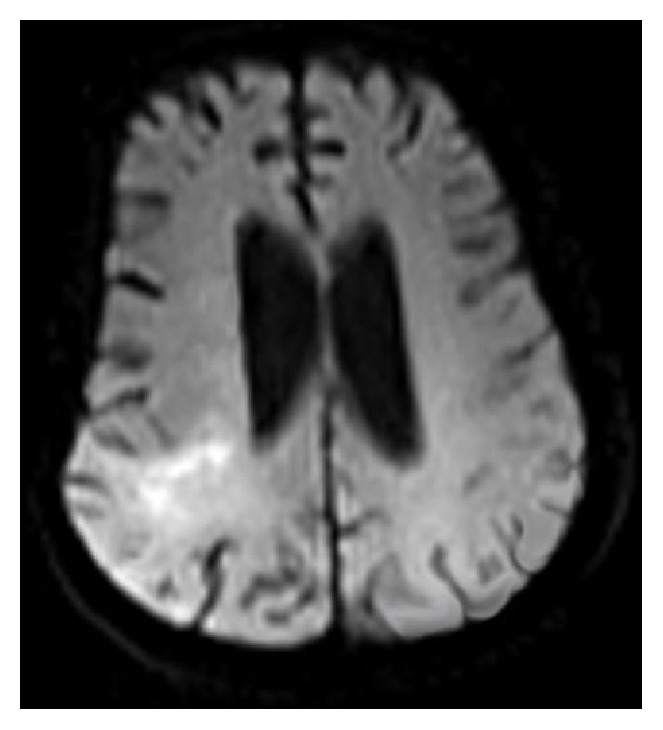
Axial DW image showing tumor (glioma) in area 5, axial slice 12.

The axial DW image of a subject showing tumor (glioma) in area 5, axial slice 12, is shown in Figure 4.

**Figure 5:**
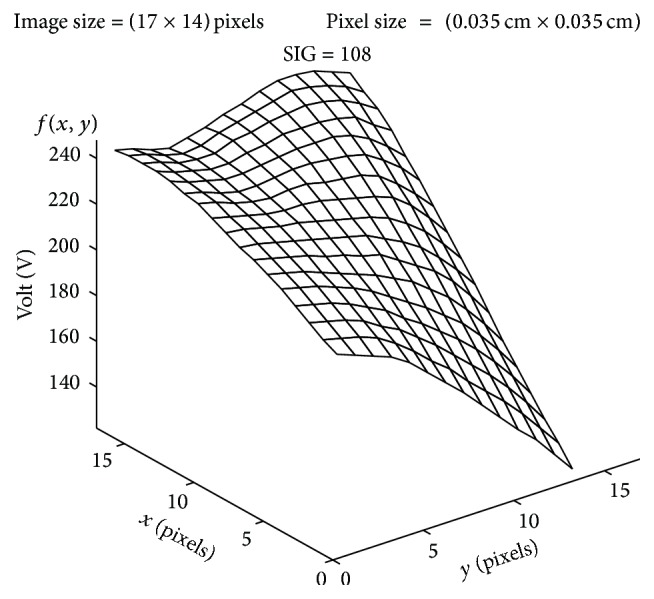
Image intensity (spatial) distribution of the subregion resulting in maximum SIG on tumor side (area 5, axial slice 12).

**Figure 6:**
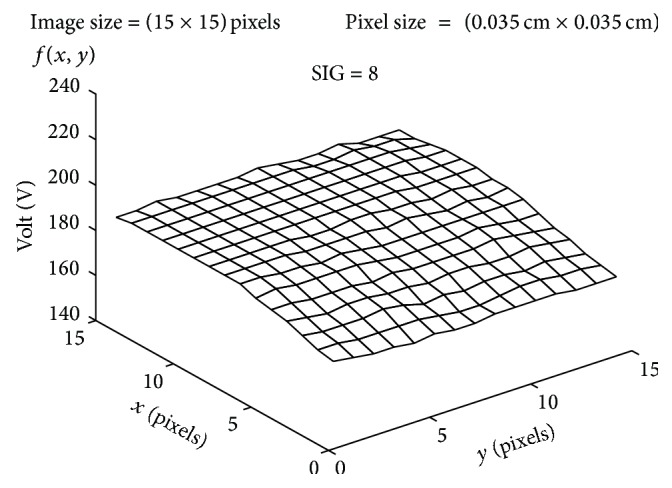
Image intensity (spatial) distribution of the subregion resulting in minimum SIG on contralateral side.

The plot of the spatial variation of image intensity distribution of the subregion of the ROI (area 5, axial slice 12) resulting in maximum SIG value (resultant SIG on tumor side), for the subject shown in Figure 4, is shown in Figure 5. The plot of the spatial variation of image intensity distribution of the subregion on the contralateral hemisphere resulting in minimum SIG value (resultant SIG on contralateral side) for the same subject is shown in Figure 6.

It is observed from Figure 5 that the spatial intensity variation distribution of the subregion on the tumor side has abrupt jumps in contrast to the spatial intensity variation distribution of the subregion on the contralateral hemisphere, which is almost unvarying as shown in Figure 6. The nonuniform image intensity distribution in the subregion on the tumor side results in a higher value for the SIG parameter (108). In contrast, the uniform intensity distribution in the subregion on the contralateral hemisphere results in a considerable smaller value for the SIG parameter (8). The resultant SIG value evaluated from the subregion of the ROI on the tumor side indicates the maximum change in the signal intensity variation across the ROI on the axial DW slice.

**Figure 7:**
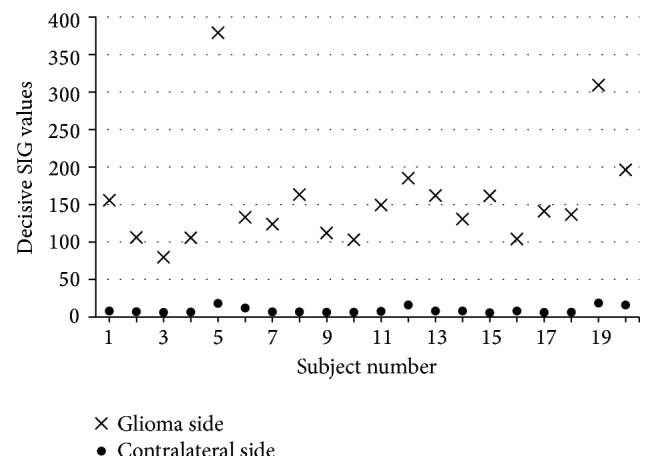
Variation in the decisive SIG values on glioma side and the corresponding decisive SIG values on the contralateral side for the glioma subjects.

**Figure 8:**
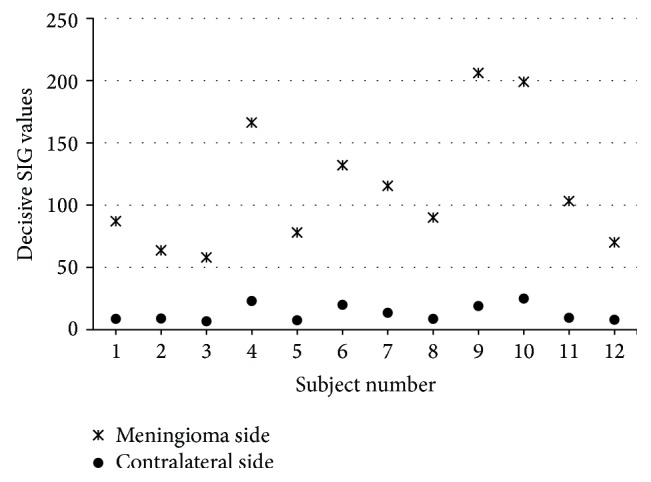
Variation in the decisive SIG values on meningioma side and the corresponding decisive SIG values on the contralateral side for the meningioma subjects.

The plot of the variation in the maximum SIG values (decisive SIG value on tumor side) on the glioma side and the minimum SIG values (decisive SIG values on contralateral side) on the contralateral hemisphere, for all the 20 subjects with glioma, is shown in Figure 7. Similarly, the plot of the variation in the decisive SIG values on the meningioma side and the corresponding decisive SIG values on the contralateral hemisphere, for all the 12 subjects with meningioma, is shown in Figure 8.

Statistical study is carried out by using the Excel program and SPSS 17.0 software package. The analysis is performed at 99% confidence level (*P* < 0.01) using the significance of difference between the means using Student's *t* distribution [40]. The decisive SIG values for the subjects with glioma and meningioma are statistically compared to the corresponding values from the contralateral hemisphere. The difference in the decisive SIG values for the tumor subjects compared to their contralateral hemisphere are highly significant (*P* < 0.01) in the regions of the brain, where there was a high incidence of tumor (glioma/meningioma). Results suggest that the decisive SIG values obtained from DW images for the subjects with glioma or meningioma can be used to clearly differentiate the tumor subjects from the normal subjects.

**Figure 9:**
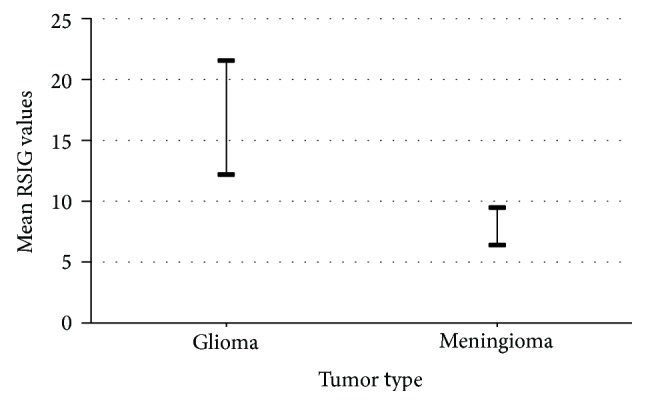
Variation in the mean RSIG values with the tumor types.

The results of analysis for the subjects with glioma and meningioma considered in the present study are summarized in Table 1. The range of the decisive SIG values obtained for the subjects with glioma and meningioma and their corresponding decisive SIG values on the contralateral hemisphere are listed. Also the range of the RSIG values evaluated across the subjects with glioma and meningioma is listed. The plot of the variation in the mean RSIG values across the subjects with glioma and meningioma (last column of Table 1) is shown in Figure 9. It is observed that the mean RSIG value obtained for the subjects with glioma (16.87 ± 4.69) is notably higher in comparison to the mean RSIG value for the subjects with meningioma (7.94 ± 1.54).

The statistical significant difference between the RSIG values obtained across the two tumor types is tested. Statistical study is carried out at 99% confidence level (*P* < 0.01) using the significance of difference between the means using Student's *t* distribution [40]. It is observed that the mean RSIG value is significantly different (*P* < 0.01) across the subjects with glioma and meningioma (as listed in the last column of Table 1).

Our study is primarily based on quantifying the signal intensity variations observed on DW images for the differential diagnosis of glioma and meningioma. The variation in the signal intensity observed in the area of these brain tumors does not depend on the size/shape and the location of these tumors. However, these details might play an important role in the further planning of management for the tumor subjects. On the other hand, as the signal intensity variations depend on the tumor stage (higher grade or more aggressive tumors leading to greater signal intensity variations), the results obtained in the study largely depend on the tumor stage.

The method proposed in the present work is realized with a purpose to overcome the drawbacks [18, 28–30] associated with the studies reported in literature for measurements using ADC methods. The proposed method speeds up the process of evaluating the brain tumor as it can be carried out routinely, without relying on a dedicated workstation to make the measurements, as in ADC methods. It adopts a standardized way to place the ROI to cover the entire volume of the tumor on the axial DW slice and thereby provides quantitative information about the tissue characteristics and heterogeneity of the whole tumor. Further, in our method considering the entire area of the tumor eliminates the requirement of expertise, as selection of a localized area within a tumor can be subjective and prone to sampling bias [31]. Also, all the affected axial DW slices are taken into account in order to reach the results, in contrast to the ADC methods, wherein, a single axial DW slice (showing maximum intensity changes) is considered in evaluating ADC values. In our method taking multiple axial DW slices additionally guarantees that there is minimum loss of information, as larger area of the brain is covered for making observations about the tumor. Therefore, the work presented here provides a useful method that is both quantitative and user-friendly, with a direct measurement of signal intensity on DW images, based on the entire tumor volume (all the DW axial slices considered) that can be positively useful in differentiating between subjects with glioma and subjects with meningioma.

## 4. Conclusions

Analysis of the results suggests that the SIG values on DW images in the region of brain tumor can be used to markedly distinguish the tumor subjects quantitatively from the normal subjects. Comparing the relative increase in the SIG values (RSIG) on DW images for all the subjects with glioma and meningioma, it is found that they are in the range of (10.08–28.36) times and (5.60–9.86) times, respectively, compared to their contralateral normal hemisphere. The quantitative changes in the RSIG values based on the entire tumor volume can be assessed and can be positively useful in differentiating between subjects with glioma and subjects with meningioma. Therefore the adoption of the quantitative method indicated here, in the clinical diagnosis of brain tumor, could be useful and informative.

## Figures and Tables

**Table 1:** Decisive SIG and Mean RSIG values for subjects with glioma and meningioma.

Tumor type	Number of cases	Decisive SIG values (*x* ± *s*)		Mean RSIG values (*x* ± *s*)
		Tumor side	Contralateral side	
Glioma	20	156.75 ± 71.42	9.17 ± 4.33	16.87 ± 4.69
Meningioma	12	114.05 ± 51.41	13.22 ± 6.67	7.94 ± 1.54

^*^(*x* ± *s*) = (mean ± standard deviation).
